# High-wearable EEG-based distraction detection in motor rehabilitation

**DOI:** 10.1038/s41598-021-84447-8

**Published:** 2021-03-05

**Authors:** Andrea Apicella, Pasquale Arpaia, Mirco Frosolone, Nicola Moccaldi

**Affiliations:** 1 Department of Electrical Engineering and Information Technology, University of Naples Federico II, Naples, Italy; 2 Department of Public Health and Preventive Medicine, University of Naples Federico II, Naples, Italy; 3 Interdepartmental Center for Research on Management and Innovation in Healthcare (CIRMIS), University of Naples Federico II, Naples, Italy

**Keywords:** Attention, Rehabilitation, Computer science

## Abstract

A method for EEG-based distraction detection during motor-rehabilitation tasks is proposed. A wireless cap guarantees very high wearability with dry electrodes and a low number of channels. Experimental validation is performed on a dataset from 17 volunteers. Different feature extractions from spatial, temporal, and frequency domain and classification strategies were evaluated. The performances of five supervised classifiers in discriminating between attention on pure movement and with distractors were compared. A *k-Nearest Neighbors* classifier achieved an accuracy of 92.8 ± 1.6%. In this last case, the feature extraction is based on a custom 12 pass-band Filter-Bank (FB) and the Common Spatial Pattern (CSP) algorithm. In particular, the mean Recall of classification (percentage of true positive in distraction detection) is higher than 92% and allows the therapist or an automated system to know when to stimulate the patient’s attention for enhancing the therapy effectiveness.

## Introduction

Ang et al. prove that a neuromotor rehabilitation exercise induces neuronal neuroplasticity and promotes motor recovery^1^. In particular, the repetition of the exercise induces a reorganization of the motor cortex. However, the repetition of the same exercise may induce weariness in the subject and prevent a careful focus on the performance of the exercise. Conversely, completing the exercise, while maintaining the attention focus in a sustained and selective way, promotes neuronal plasticity and motor learning^2,3^. The attention to the motor task has an enhanced effect on rehabilitation performance^4^.

Ladvas and Berti describe attention as the function that regulates the filtering and organization of the information received from a subject, allowing his/hers adequate responses^5^. Sohlberg and Mateer propose a characterization of attention in four different dimensions^6^: (i) the *Arousal* indicates the activation level and defines the psychophysiological activation allowing the afference of the different stimulations; (ii) the *Selective attention*: points out the ability to focus attention on a specific source or sensory channel; (iii) the *Distributed attention* is the ability to simultaneously process information from multiple sources; and (iv) the *Sustained attention* is the ability to direct and maintain cognitive prolonged activity on a specific stimuli.

In everyday life, many types of distracting effects (visual, auditory, and their combinations) sidetrack attention when performing any task, especially if it requires engagement^7^. Diez et al. identified attention just as the ability to select interesting stimuli, by ignoring other distracting stimuli in the surrounding environment^8^. These distractors play a fundamental role in analyzing the attentional process^9^. Changes in cognitive processes related to attention activate different parts of the brain. Concurrent distracting events deactivate certain brain areas by activating other ones^10^. The use of distracting stimuli during the execution of a motor task, as opposed to the careful concentration, characterizes the experimental set-ups of the studies on the measurement of motor attention^7,11^. Many studies deal with assessing the attention and its different dimensions through the analysis of the brain signals using the ElectroEncephalography (EEG)^12^. EEG is the most used technique because of its high temporal resolution, non-invasiveness, and low cost. Several studies have shown that the level of attention affects the EEG signal^13,14^. Therefore, variations in the EEG signal can be used to detect corresponding changes in attention levels^15^. Attention creates a variation in brain signals that can be assessed both in the time and in the frequency domain^16^.

Most of the studies in the rehabilitation sector adopted a within subject approach for training the classifiers in distraction detection. Asayb et al. in 2017^7^ proposed to assess the attention during the flexion-extension of the ankle in presence of auditory distractors. Using a 18-channel system and wet electrodes on 12 participants, they obtained an average accuracy of 71%, by extracting time-frequency features from 1.5-s epochs. Hamadicharef, Brahim et al.^17^ proposed an interesting processing system (already widely used in the EEG field for Motor Imagery) for assessing the attention, during a cognitive task with eyes closed and opened. This processing involves a Filter-Bank in relation to the Common Spatial Pattern. A 15-channel EEG system achieves an average accuracy of 69.2% on five subjects with a 2-s time window. Antelis et al.^18^ proposed the distraction detection during robot-assisted passive movements of the upper limb. Six patients were connected to a 32-channels EEG by wet electrodes and to the robot’s end-effector for assisted passive movements. They got an average accuracy of 76.37% in classifying 3-s epochs, when mentally count back in threes, starting in a self-selected random three-digit number, assured the distraction condition. In 2019 Asayb et al.^11^ proposed an upgrade of their previous work using a 28-channel EEG system and wet electrodes. Three different distractors characterized the experimental set-up. Signal processing was based on spectro-temporal features extracted from 3-s epochs. The obtained average accuracy was 85.8% by exploiting the motor-related cortical potential. However, in this state of the art, an appropriate approach for clinical application seems to be missing. The high number of channels and the use of wet or semi-wet electrodes penalize the wearability, limiting the clinical usability. In this paper, an EEG-based method to detect the lack of focused (selective and sustained) attention during the execution of a neurorehabilitative motor task is proposed. The EEG signal is measured by a wearable, non-invasive system, with a very-small number of dry electrodes. A state-of-the-art accuracy is achieved in classifying 3 s epochs. In particular, in “Proposal” section, the basic ideas and the data analysis of the proposed method are illustrated. Then, in “Experimental validation results” section, the experimental validation is reported, detailing the laboratory test procedure and discussing the comparison results of a feature extraction and classification.

## Proposal

### Basic ideas

The proposed method for detecting distraction during motor rehabilitation is based on the following key concepts:

- *EEG-based distraction detection*: During a rehabilitation motor task, EEG trend is influenced by the state of the patient attention or distraction to the task itself.
- *Attention vs distraction definition*: Focusing on motor task means imagining, with open eyes, the movement while its execution and trying not to think about anything else. A distracting condition occurs when the patient performs an entirely absorbing cognitive task while continuing to carry out the rehabilitation movement. To the end of evaluating the phenomenon, a rehabilitative motor task is carried out. The assignment is run under conditions of concentration on the action and in the presence of a distractor (auditory, visual, and visuo-auditory) which engages the learner in a concurrent cognitive task analogously as what done in Asayb et al.^11^.
- *Metrology perspective*: An applied metrological and instrumentation-aimed approach is guaranteed, for the first time, in the EEG based distraction detection.
- *Feature extraction enhancement*: After an artifact removal performed by an Independent Component Analisys (ICA) based algorithm, a multiple bandpass Filter-Bank, in combination with a Common Spatial Pattern algorithm, selects spatial, temporal and frequency features. In particular, a 12-band Filter-Bank is proposed for enhancing, the peculiar contribution of the delta, theta, and alpha bands as fundamental in the analysis of attentional processes^19^, compared to previous 9-band approaches^17^.
- *High wearability*: The EEG acquisition system is realized in ultra-light foam. The ergonomic and comfortable device is equipped with a rechargeable battery and transmits the acquired data via Bluetooth. Dry electrodes avoid the inconvenient of electrolytic gel.
- *Clinical applicability*: wearability cannot be a prejudice for accuracies compatible with clinical use. A method with state-of-the-art accuracy (greater than 80%^11,17^) is required.
- *Validation based on wide comparison*: Performance of the proposed method are compared with different strategy of EEG feature extraction (including the proposal of Hamadicharef et al.^17^), and different types of classifiers.

### Method

**Figure 1:**
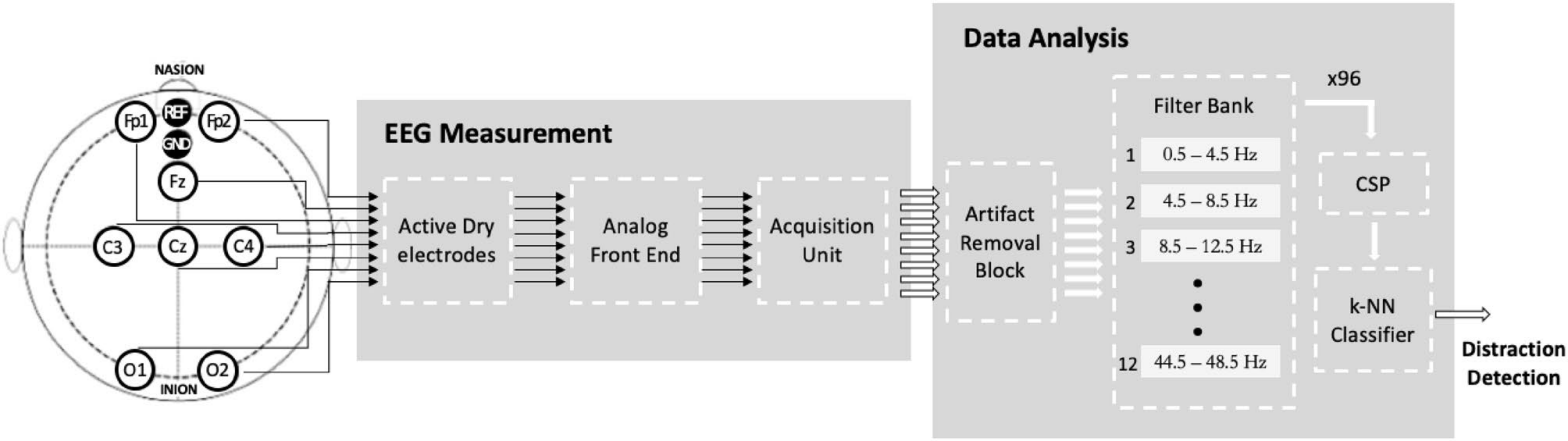
The proposed distraction-detection method (CSP: Common Spatial Pattern algorithm).

The proposed method is depicted in Fig. 1. The EEG signals are acquired by *Active Dry Electrodes* from the scalp. Each channel is differential with respect to AFz (REF), and referred to Fpz (GND), according to 10/20 international system. Analog signals are first transduced by the *Active Dry Electrodes* and then conditioned by the *Analog Front End*. Next, they are digitized by the *Acquisition Unit* and transmitted to the *Data Analysis* stage. Here, after an artifact removal performed by an ICA based algorithm, suitable features are extracted by the chain of a 12-component *Filter Bank* and a *Common Spatial Pattern* (CSP) algorithm. Then, a classifier receives the feature arrays and detects distraction.

### Data analysis

In this section, (i) the *feature selection and extraction* and (ii) the *classification * are detailed.

#### Feature selection and extraction

The EEG signal, acquired through eight channels, was filtered through a 12 IIR band-pass Filter Chebyshev type 2 filter bank, 4 Hz amplitude, equally spaced from 0.5 to 48.5 Hz. In Hamadicharef et al.^17^, a filter bank with 9 filters of 8 Hz amplitude equal to [0–40] Hz, with a 4 Hz overlap, was proposed. This solution subdivided the traditional EEG beta and gamma bands into sub-bands, however combining other bands (delta and theta with the first filter between 0 and 8 Hz, as well as theta and alpha with the second filter between 4 and 12 Hz). Considering the relevance of the delta, theta and alpha bands in the analysis of the attention highlighted in Graber et al.^20^ and in Coelli et al.^19^, the solution proposed in this study allows to enhance their peculiar contribution.

The unit of analysis of the classification activity was identified in time windows of 3 s with an overlap of 1.5 s. Considering a sampling frequency of 256 Sa/s, each of these record is therefore composed of 96 EEG tracks (obtained by applying the 12 filters of the Filter Bank on each of the 8 channels), each one of 1536 samples.

A Common Spatial Pattern (CSP) was used as a spatial filtering algorithm. CSP is one of the most used feature extraction methods for classifying EEG signals^17,21^. In a binary problem, the CSP acts by calculating the covariance matrices relating to the two classes. These two matrices are simultaneously diagonalized in a way that the eigenvalues of two covariance matrices sum up to 1. Through the subsequent use of a bleaching matrix, a suitable projection matrix is identified in order to reorganize the input into a number of components consistent with the dimensions of the input matrix. In a binary problem, these components are sorted on the basis of variance in order: (i) *decreasing*, if the projection matrix is applied to inputs belonging to class 1, and (ii) *ascending*, in case of inputs belonging to class 2^22^. In this study, the CSP receives the records (epochs) as 3D tensors (channels, filters, and samples). It outputs 2D matrices (channels, filters) reducing the dimensionality of the features by a factor of 1536 (number of sample).

#### Classification

A k-Nearest Neighbour (*k*-NN) classifier is used for classifying the CSP output. Compared to other supervised machine learning methods, *k*-NN is a non-parametric method (i.e., without a priori assumption on the data) which uses the labelled data itself for the classification without any training. The behavior of *k*-NN in its simplest version can be described as follows: given a set *D* of labelled points, a distance measure (e.g., Euclidean, Minkowski) and a positive integer *k*, when a new unlabelled point *p* is presented, the *k*-NN algorithm searches in *D* for the *k* points nearest to *p*, so the most present class label along its *k* neighbors is assigned to *p*. Thus, the only hyperparameters required to *k*-NN are a positive integer *k* and the distance measure to use together with any parameters related to the distance measure if needed. These hyperparameters were set using a cross-validation procedure. *k*-NN has already been widely used in EEG signal analysis showing interesting results (see for example^23^).

## Experimental validation results

In this section, the experimental assessment of our proposal is reported and the results are discussed.

### Experimental protocol

**Figure 2:**
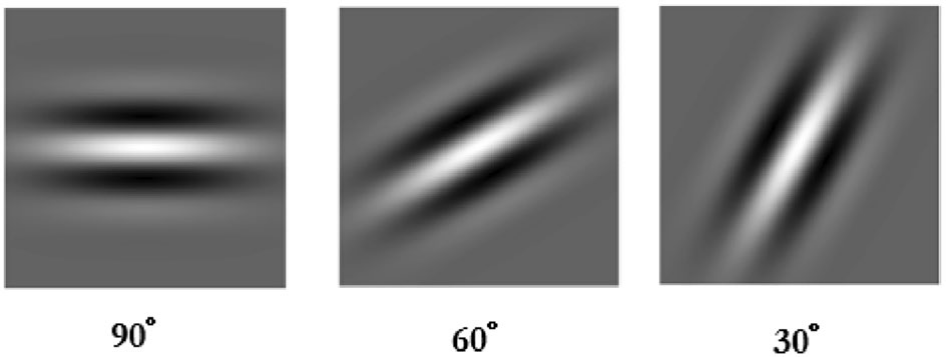
Visual Distractor task elements based on visual Gabor mask with different orientation: 90$^\circ$, 60$^\circ$, and 30$^\circ$.

The ethical committee approved the experimental protocol of the University of Naples Federico II. A written informed consent was obtained from each volunteer before the experiment. All experiments were carried out in accordance with relevant guidelines and regulations. A session was based on seventeen volunteers subjects (eleven males and six females, with an average age of 30.76 ± 8.15). All of them had a normal clinical history with normal vision and normal hearing, and no neurological disease. The participants were seated in a comfortable chair with armrests, in a very quiet room, about one meter away from a PC screen. After wearing the EEG-cap, participants were requested to execute a squeeze-ball exercise whenever a start command appeared on the PC screen. Squeeze-ball is one of the most common hand rehabilitation exercises^24^. Following a period of immobilization in plaster, after a surgical intervention or in the presence of inflammatory or degenerative pathologies (e.g., arthrosis, rheumatoid arthritis), hand-ball rehabilitation showed to be important in maintaining or restoring the functional use of the hand^25^. Motor task execution consists of maintaining attention focused only on: (i) the squeeze movement (*attentive-subject trial*), or (ii) a concurrent distractor task (*distracted-subject trial*); in both trials the participant must perform the squeeze-ball movement. An aneroid sphygmomanometers supported the user attention to motor task execution: volunteers were asked to focus the aneroid gauge, while squeezing the bulb and pumping air into the cuff. The distractor task was based on the *Oddball paradigm*^26,27^: the presentations of sequences of repetitive stimuli, infrequently interrupted by a deviant stimulus. The oddball paradigm is one of the most widely used methods to study the neurophysiology of attention. In the proposed protocol, the volunteer was asked to count the number of certain stimuli sequences. Three types of stimuli sequences were proposed: (i) acoustic, played with a conventional headphone, (ii) visual, displayed on a PC screen, and (iii) and visual-aucoustic combination^28^. Each participant completed one session composed of 30 trials: 15 *attentive-subject trial* and 15 *distracted-subject trial*. The trials sequences were randomly chosen for minimizing the influence of task learning. Each trial consisted of: 2 s task presentation, 9.5 s task execution and 5 s relax. Furthermore, a 15 s baseline was acquired at the beginning of the session. In the following, trial contents are detailed:

- *Attentive-subject trial* An Attentive-subject trial notification appears for 2 s on the PC screen. Then, a ball-squeezing image triggers the start of the motor exercise and a new message on the screen asks the subject to focus on the squeezing movement. At the end of the task execution, an image of a relaxing landscape is shown for 5 s.
- *Distracted-subject trial* A notification concerning the distractor task (Audio, Visual or Audio-Visual) appears for 2 s on the PC screen. Then, an acoustic message notices the beginning of the motor exercise; a distractor task (based on Oddball paradigm), chosen among the followings, starts:
  - The *Audio Distractor * is based on the auditory oddball paradigm. Eight tones sequences sound through the earbuds. Tones range among three different frequencies: *low*, 500 Hz, *middle*, 1200 Hz, and *high*, 1900 Hz. The tone *low* has 50% probability of occurrence. The occurrence probability of the *middle* and the *high* tones is 25%. The target sequence is the appearance of a diverted tone after the other more frequent one: when the *middle* tone occurs immediately after the *low*, or when the *high* occurs immediately after the *low*. Others combinations are not considered as target occurrences.
  - The *Visual Distractor* task is based on the visual oddball paradigm. Three 2D-Gabor masks were used with different orientation: 90°, 60°, and 30° (Fig. 2). The 2D-Gabor mask is a Gaussian kernel function modulated with sinusoidal plane wave. The most probable Gabor (50% of probability) has orientation of 90° , while the diverted Gabor (25% of probability) has 60° or 30° orientation. Eight Gabor sequences occurred on the PC screen. The target sequence was the occurrence of diverted Gabor mask (with orientation of 60° or 30°) after the most frequently with 90° orientation.
  - The *Audio-Visual Distractor* task is a combination of the previous oddball paradigms. Eight between tone and Gabor sequences occur randomly. The target sequence is the occurrence of any Gabor mask after the tone. Others combination sequences are not target occurrences.
  At the end of the task, a relaxing landscape is presented for 5 s. During the relax period, the subjects are asked to give the number of the observed targets.

### EEG instrumentation

**Figure 3:**
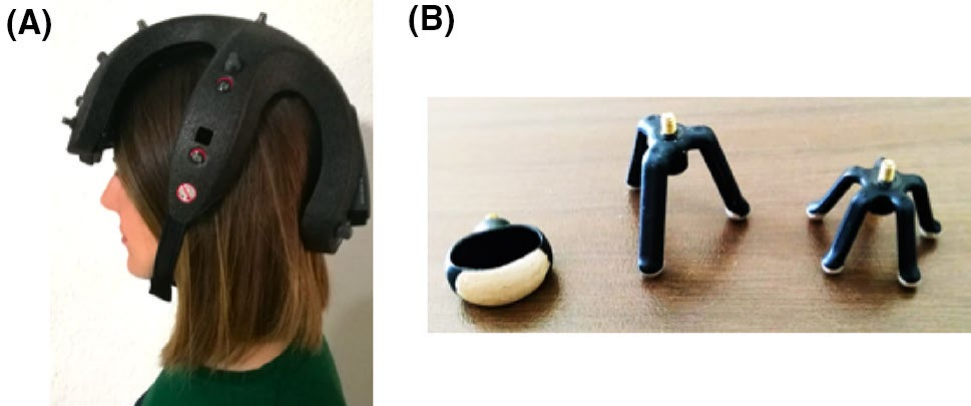
(**A**) EEG data acquisition system *Helmate8*, and (**B**) Different configuration of dry electrodes from *abmedica*.^29^.

In this study, the commercial EEG acquisition system *AB-Medica Helmate*^29^ is employed (Fig.3A).

The device, composed of ten dry electrodes, guarantees eight acquisition channels. The EEG signal is acquired by dry electrodes made of conductive rubber with an Ag/AgCl coating at their endings^30^. Three different types of electrodes, with different shapes, are used to pass hair and reach the scalp or join to the hairless areas (Fig. 3B). The output signal is recorded as difference between each of 8 channels and the ground electrode (Fpz)^31^. Then, the difference is referenced with respect to the electrode (AFz). A dedicated software (*Helm8 Software Manager*) allows to check the contact impedance between the electrodes and the scalp. EEG signal is acquired with a sampling rate of 512 Sa/s. The acquisition software allows to use several filters (e.g., notch and IIR). This data acquisition system is a certified EEG system Class IIA (according to Medical Device Regulation (EU) 2017/745) with accurate components. A Texas Instruments analog front-end, the ADS1298^32^ with a 24-bit, $$\Delta$$$$\Sigma$$ analog-to-digital converter (ADCs) with built-in programmable gain amplifiers (PGAs), internal reference, and an onboard oscillator, are exploited. The device exhibits the following main metrological performances: (i) CMRR: -115 dB; (ii) eight low-noise PGAs and eight high-resolution ADCs (ADS1298, ADS1298R); (iii) input-referred noise: 4 $$\mu$$VPP (150 Hz BW, G = 6); and (iv) input bias current: 200 pA; joined to the following operating performances: (i) low power: 0.75 mW/channel; and (v) data rate: 250 Sa/s to 32 kSa/s.

### Data processing

During the experiments 4590 epochs composed of 8 channels of 512 samples were acquired. In Table 1 number of (i) subjects, (ii) sessions, (iii) trials, (iv) epochs per trial (v) epochs per subject, and (vi) epochs as a whole are reported.

**Table 1:** Data-set composition.

# Subjects	# Sessions	# Trials per session	# Epochs per trial	# Epochs per subject	# Total epochs
17	3	30	3	270	4590

Half of the epochs were collected during the *attentive-subject trials* and were labeled as belonging to the first class. The remaining part was acquired during the *distracted-subject trials* and was labeled as belonging to the second class. The recorded EEG was divided in 3 s epochs. Each epoch was filtered between 0.5 and 48.5 Hz using a zero-phase 4th-order digital butterworth filter. An independent component analysis (ICA) algorithms—Infomax-ICA^33^—filtered out artifacts from the signal. In particular the version implemented by *Runica* module of *EEGlab* tool was adopted. Feature extraction was implemented either in time domain and frequency domain. For the latter Relative and Absolute Power Spectral Density at varying of frequency bands were considered. Three different frequency bands articulation were examined:

- seven traditional EEG bands: delta [1–4] Hz, theta [4–8] Hz, alpha [8–12] Hz, low beta [12–18] Hz, high beta [18–25] Hz, low gamma [25–35] Hz, and high gamma [35–45] Hz; in this case, the number of features for each epoch was 112 (7 bands * 2 PSD (relative and absolute) * 8 channels);
- nine 8 Hz bands, 4 Hz overlapped, in the range [1–40] Hz; the number of features for each epoch was 144 (9 bands * 2 PSD (relative and absolute) * 8 channels);
- twelve 4 Hz bands, non-overlapped, in the range [0.5–48.5] Hz; the number of features for each epoch was 192 (12 bands * 2 PSD (relative and absolute) * 8 channels);

Regarding time domain, the feature extraction was based on four different approaches:

- only CSP: in this case, the number of features for each epoch was 8 (CSP remaps the input information in a new space with dimensionality equal to the number of channels);
- CSP preceded by different types of Filter-Banks: three different types of Filter-Banks were applied with the same band articulation proposed for the feature extraction in the frequency domain. In these cases CSP remaps the input information in a new space having dimensionality equal to the number of channels (8) multiplied with number of bands, obtaining 56, 72, and 96 number of features respectively.

Five supervised machine learning binary classifiers were used for discriminating between attention or distraction conditions: k-Nearest Neighbour (k-NN), Support Vector Machine (SVM)^34^, Artificial Neural Network (ANN)^34^, Linear Discriminant Analysis (LDA)^35^, and Naive Bayes (NB)^36^. Regularization terms were exploited in the training procedures for neural networks and SVM learning processes, using a weight decay and the soft-margin formulation, respectively. All the classifiers were tested on the seven features types described above. For each subject, the hyperparameters of each classifier were selected by a random search with Nested Cross Validation to mitigate possible bias induced by the low sample size^37^. Differently from the classical *k*-fold cross validation, Nested CV is composed of two nested *k*-fold cross validation procedures: the inner one finds the best model hyperparameters, and the outer one estimates the performance of the inner search. Namely, in the classic *k*-fold CV, given a combination of the hyperparameters values, a set of data is divided into a partition of *k* subsets (folds). Thus, a set $T_I$ composed of $k-1$ folds is used to train the model and the remaining fold $E_I$ is used for the performance evaluation by computing the appropriate metric scores (e.g., accuracy). This process is repeated for all the combinations of the *k* folds, by making different pairs of training set $T_I$ and test set $E_I$ at each iteration. In this way, final average metrics scores between all the different test sets $E_I$ are computed. This process is then repeated for each hyperparameters combination, finally returning the best average metrics values together with the related hyperparameters. In this process, the model is evaluated together with the hyperparameters tuning. Instead, in the nested cross validation CV procedure, an outer CV makes a first division of the data into *l* folds; then, a set $T_O$ composed of $l-1$ folds is used as input to a classical inner *k*-fold CV procedure, as above described (and therefore further divided into *k* folds by the inner CV procedure). Then, the returned best hyperparameters values are used to train the model on the $T_O$ set as a whole and tested on the remaining fold, say $E_O$. This process is repeated for all the combinations of the *l* folds and the final average metrics on the $E_O$ sets are reported. In this way, the nested CV process avoids a possible bias on the model, due to the use of the same data for the model hyperparameters tuning and the model evaluation. In this study, a tenfold Nested CV was used. In the outer layer, 10% of the data was separated for test and the rest of the data was used to develop a model. In the internal layer, the remaining 90% of the data was used for tuning the hyperparameters. Training and test sets were obtained without separating the trials consisting of 3 epochs each. In this way, the training and the test sets do not include parts of the same trial. The hyperparameters variation range are displayed in Table 2.

**Table 2:** Classifier optimized hyperparameters and variation range.

Classifier	Hyperparameter	Variation range
k-nearest neighbour (k-NN)	Distance (DD)	{Cityblock, chebychev, correlation, cosine, euclidean, hamming, jaccard, mahalanobis, minkowski, seuclidean, spearman}
	DistanceWeight (DW)	{Equal, inverse, squaredinverse}
	Exponent (E)	[0.5, 3]
	NumNeighbors (NN)	[1, 5]
Support Vector Machine (SVM)	BoxConstraint (BC)	Log-scaled in the range [1e−3, 1e3]
	KernelFunction (KF)	{Gaussian, linear, polynomial}
	KernelScale (KS)	Log-scaled in the range [1e−3, 1e3]
	PolynomialOrder (PO)	{1,2,3,4}
Artificial Neural Network (ANN)	Activation Function (AF)	{relu, sigmoid, tanh}
	Hidden Layer nr. of Neurons (HLN)	[25, 200]
Linear Discriminant Analysis (LDA)	Gamma (G)	[0,1]
	Delta (D)	Log-scaled in the range [1e−6, 1e3]
	DiscrimType (DT)	{Linear, quadratic, diagLinear}
		{diagQuadratic, pseudoLinear, pseudoQuadratic}
Naive Bayes (NB)	DistributionName (DN)	{Normal, kernel}
	Width (W)	Log-scaled in the range [1e−4, 1e14]
	Kernel (K)	{Normal, box, epanechnikov, triangle}

### Experimental results

A within-subjects approach was realized. The accuracy (mean and standard deviation) for each classifier was assessed at varying the type of input feature. Table 3 shows better performances in case of features extracted from the time domain by combining Filter-Bank and CSP.

**Table 3:** Within-subject accuracy (mean and standard deviation percentage of the 17 subject accuracy) at varying feature and classifier.

Classifier	Feature						
	Frequency domain			Time domain			
	7 Traditional EEG bands	9 EEG bands proposed in^17^	Proposed 12 EEG bands	CSP	Filter-Bank + CSP		
					7 Traditional EEG Bands	9 EEG bands proposed in^17^	Proposed 12 EEG bands
*k*-NN	77.5 ± 5.5	76.7 ± 5.5	80.2 ± 5.1	65.9 ± 5.0	87.4 ± 4.1	90.9 ± 3.2	**92.8 **±** 1.6**
SVM	79.9 ± 5.6	76.0 ± 4.0	81.7 ± 6.9	69.2 ± 5.1	86.8 ± 4.5	89.8 ± 3.7	91.1 ± 3.2
LDA	76.7 ± 7.4	75.1 ± 7.2	78.3 ± 6.3	67.7 ± 4.8	82.9 ± 4.5	85.7 ± 6.2	86.6 ± 2.0
ANN	75.6 ± 6.3	73.6 ± 6.7	76.9 ± 6.4	67.2 ± 4.5	81.9 ± 4.5	85.1 ± 5.0	86.3 ± 3.5
NB	64.5 ± 6.2	63.8 ± 5.2	65.3 ± 7.8	65.2 ± 4.9	75.3 ± 7.3	77.0 ± 7.2	78.7 ± 7.5

The best performance value is highlighted in bold.

**Figure 4:**
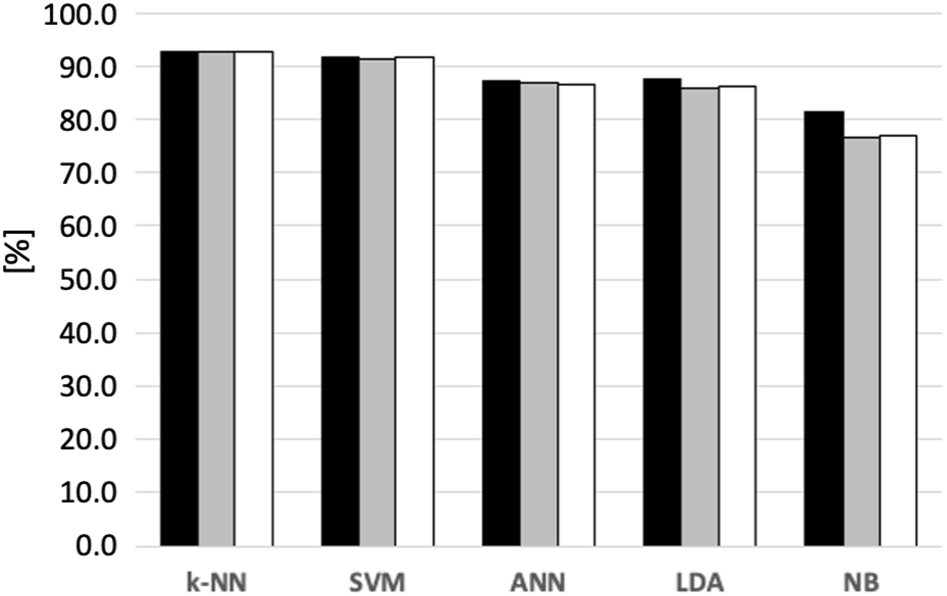
F-Measure test results for the best performance of each classifier: Precision (black) , Recall (gray), and F1-score (white).

In particular, the proposed solution based on 12 bandpass Filter-Bank provides the best performances for all classifiers except for LDA. In Table 4, the accuracy of the proposed solution is shown for each subject at varying the classifier. In case of k-NN, the mean accuracy reached the maximum value of 92.8 ± 1.6%. To the best of the authors’ knowledge, the accuracy obtained can be considered state-of-the-art when considering a within subjects approach. Regarding rehabilitation goals, the minimization of failure in recognizing distraction is the main issue. Therefore, an F-measure test was carried out to assess the classification performance in minimizing false negatives for the second class (distraction) analysis. Figure 4 shows a k-NN mean Recall higher than 92%.

**Table 4:** Within-subject accuracy of the proposed solution based on the 12 bandpass Filter Bank and Common Spatial Pattern at varying the classifier.

Subject	Classifier				
	k-NN	SVM	LDA	ANN	NB
#1	91.1 ± 5.3	90.3 ± 5.2	88.2 ± 5.3	86.3 ± 7.2	66.0 ± 9.7
#2	92.2 ± 2.2	90.1 ± 5.1	85.1 ± 6.2	83.5 ± 5.3	79.2 ± 9.9
#3	93.3 ± 5.5	92.2 ± 5.1	89.2 ± 7.1	80.3 ± 7.3	87.2 ± 7.3
#4	94.1 ± 4.2	95.0 ± 2.2	89.6 ± 5.5	92.4 ± 6.8	81.3 ± 4.4
#5	90.4 ± 4.3	89.2 ± 6.7	84.3 ± 9.2	84.5 ± 7.6	65.3 ± 9.7
#6	93.3 ± 3.1	96.5 ± 3.8	91.7 ± 6.8	89.7 ± 6.2	74.1 ± 7.3
#7	96.1 ± 3.2	92.3 ± 4.4	87.2 ± 6.8	87.6 ± 8.3	80.0 ± 9.8
#8	93.1 ± 5.2	91.2 ± 6.7	88.4 ± 7.3	87.6 ± 6.1	86.5 ± 6.3
#9	91.2 ± 4.5	89.1 ± 8.8	88.4 ± 9.1	87.6 ± 6.5	82.8 ± 6.2
#10	92.1 ± 4.4	85.2 ± 4.8	80.3 ± 5.7	82.3 ± 6.9	73.2 ± 9.9
#11	91.1 ± 5.3	90.2 ± 6.7	83.5 ± 8.5	82.5 ± 9.1	79.2 ± 7.1
#12	94.8 ± 4.2	93.8 ± 3.3	91.7 ± 6.6	87.6 ± 06	87.3 ± 3.5
#13	93.3 ± 6.2	92.2 ± 7.6	84.2 ± 5.9	86.8 ± 8.4	75.6 ± 8.4
#14	96.6 ± 4.5	96.3 ± 5.3	90.8 ± 5.8	90.4 ± 6.1	86.8 ± 8.2
#15	93.8 ± 6.2	94.1 ± 4.5	88.8 ± 8.1	86.2 ± 6.5	84.4 ± 5.6
#16	93.5 ± 7.3	91.8 ± 5.5	86.6 ± 2.2	87.2 ± 5.5	82.5 ± 5.6
#17	93.2 ± 4.1	84.8 ± 6.5	77.5 ± 1.6	77.8 ± 1.1	66.4 ± 8.0
Mean	**92.8** ± **1.6**	91.1 ± 3.2	86.6 ± 2.0	86.3 ± 3.5	78.7 ± 7.5

The best performance mean value is highlighted in bold.

## Conclusion

A method to detect a state of attention and distraction during the execution of a motor act was proposed in this paper. The method shows experimentally a state-of-the-art mean accuracy of 92.8 ± 1.6% and a mean recall of 92.6%. Attention status classification is carried out on 3 s epochs. The level of performance achieved also arise from the use of a 12-filter custom Filter Bank which enhances the contributions of the significant EEG bands for attention analysis. The method guarantees high wearability because it exploits only eight dry electrodes and wireless data transmission. Therefore, the method turns out to be immediately usable in rehabilitation for offering to therapists: (i) a tool capable of assessing patients’ attention levels towards the proposed exercises; and (ii) the possibility to implement strategies that, through the recovery of attention, increase the rehabilitation effectiveness.
